# Propagating waves structure spatiotemporal activity in visual cortex of the awake monkey

**DOI:** 10.1186/1471-2202-14-S1-O8

**Published:** 2013-07-08

**Authors:** Lyle E Muller, Alexandre Reynaud, Frédéric Chavane, Alain Destexhe

**Affiliations:** 1 Unité des Neurosciences, Information et Complexité (UNIC), CNRS Gif-sur-Yvette, 91198, France; 2 Institut de Neurosciences de la Timone, CNRS & Aix-Marseille Université, Marseille, France; 3 McGill Vision Research, Dept. of Opthalmology, McGill University, Montréal, Canada

## 

Propagating waves of activity are seen in many types of excitable media, and in recent years, were found in the neocortex of anesthetized animals [1,2]. To date, however, it still remains unclear whether propagating waves appear during awake and conscious states [3,4]. One possibility is that these waves are systematically missed in trial-averaged data, because of their well-known variability from trial to trial [1].

To test this hypothesis, we developed a phase-based analysis technique, which works on a pixel-by-pixel basis in the unsmoothed data, and provides a quantiative means to distinguish between spatiotemporal forms of the population response. We then applied this to single-trial voltage sensitive dye imaging (VSDI) data, denoised specifically for this purpose [5], and in this work, we show definitively that spontaeous and stimulus-evoked propagating waves occur in the visual cortex of the awake monkey. Furthermore, when looking at the multiple visual areas within the imaging field in these experiments, we observe correlated propagations across primary and secondary visual cortex, illustrating a strong spatiotemporal organization of these waves across cortical areas.

These results demonstrate that propagating waves are systematically and reliably evoked by sensory stimulation, and suggest that they have the potential to affect large-scale information processing by generating a consistent spatiotemporal frame for neuronal interactions. The horizontal fiber network mediating these activity patterns has previously been implicated in active computational roles, as ascending input at a given point in cortex is known to affect the processing of future stimuli across the cortical plane [6,7]. In this work, we implicate these propagations in a specific functional role. These internally generated propagating waves provide a specific structure for the spatiotemporal activity in visual cortex, uniquely encoding both stimulus identity and time of presentation in the amplitude and phase of the population response [8]. With these results in mind, we go on to discuss the computational paradigms towards which our observations point, elucidating these with numerical models and further analysis.
